# *Achromobacter* spp. Adaptation in Cystic Fibrosis Infection and Candidate Biomarkers of Antimicrobial Resistance

**DOI:** 10.3390/ijms23169265

**Published:** 2022-08-17

**Authors:** Angela Sandri, Laura Veschetti, Giulia Maria Saitta, Rebeca Passarelli Mantovani, Maria Carelli, Gloria Burlacchini, Sara Preato, Claudio Sorio, Paola Melotti, Anna Lisa Montemari, Ersilia V. Fiscarelli, Cristina Patuzzo, Caterina Signoretto, Marzia Boaretti, Maria M. Lleò, Giovanni Malerba

**Affiliations:** 1 Department of Diagnostics and Public Health, Microbiology Section, University of Verona, Strada Le Grazie 8, 37134 Verona, Italy; 2 Department of Neurosciences, Biomedicine and Movement Sciences, University of Verona, Strada Le Grazie 8, 37134 Verona, Italy; 3 School of Health Statistics and Biometrics, University of Verona, Strada Le Grazie 8, 37134 Verona, Italy; 4 Department of Medicine, University of Verona, Strada Le Grazie 8, 37134 Verona, Italy; 5 Cystic Fibrosis Center, Azienda Ospedaliera Universitaria Integrata Verona, Piazzale Stefani 1, 37126 Verona, Italy; 6 Cystic Fibrosis Diagnostics Special Operational Unit, Microbiology and Diagnostic Immunology Unit, Bambino Gesù Children’s Hospital IRCCS, Piazza S. Onofrio 4, 00165 Rome, Italy

**Keywords:** *Achromobacter*, cystic fibrosis, drug resistance, virulence, biomarkers, adaptation

## Abstract

*Achromobacter* spp. can establish occasional or chronic lung infections in patients with cystic fibrosis (CF). Chronic colonization has been associated with worse prognosis highlighting the need to identify markers of bacterial persistence. To this purpose, we analyzed phenotypic features of 95 *Achromobacter* spp. isolates from 38 patients presenting chronic or occasional infection. Virulence was tested in *Galleria mellonella* larvae, cytotoxicity was tested in human bronchial epithelial cells, biofilm production in static conditions was measured by crystal violet staining and susceptibility to selected antibiotics was tested by the disk diffusion method. The presence of genetic loci associated to the analyzed phenotypic features was evaluated by a genome-wide association study. Isolates from occasional infection induced significantly higher mortality of *G. mellonella* larvae and showed a trend for lower cytotoxicity than chronic infection isolates. No significant difference was observed in biofilm production among the two groups. Additionally, antibiotic susceptibility testing showed that isolates from chronically-infected patients were significantly more resistant to sulfonamides and meropenem than occasional isolates. Candidate genetic biomarkers associated with antibiotic resistance or sensitivity were identified. *Achromobacter* spp. strains isolated from people with chronic and occasional lung infection exhibit different virulence and antibiotic susceptibility features, which could be linked to persistence in CF lungs. This underlines the possibility of identifying predictive biomarkers of persistence that could be useful for clinical purposes.

## 1. Introduction

*Achromobacter* spp. are opportunistic pathogens that can colonize the lungs of patients with cystic fibrosis (CF), causing chronic or occasional infections. In particular, chronic colonization has been associated with a decline in respiratory function, increased frequency of exacerbations and lung inflammation [1]. Although virulence factors supporting invasiveness and survival have been described (e.g., swimming motility, biofilm formation, lipopolysaccharide, type III secretion system, phospholipase C, proteases) [2,3,4,5,6,7,8,9,10,11,12,13], virulence features related to the ability of *Achromobacter* spp. to colonize the lungs of CF patients chronically or occasionally are still not fully clear.

Additionally, multidrug resistance strongly contributes to *Achromobacter* spp. persistence in CF patients. These bacteria show an innate resistance to many classes of antibiotics, especially to those relevant to CF lung infection treatment such as aminoglycosides, aztreonam, tetracyclines, penicillins and cephalosporins [2,14,15]. Moreover, clinical isolates exhibit acquired resistance, especially to β-lactams. The most active agents against *Achromobacter* spp. are, among others, trimethoprim–sulfamethoxazole, ceftazidime, piperacillin and carbapenems [16]; however, mutations of genes related to antibiotic resistance may occur, causing resistance to these antibiotics [13]. Indeed, isolates from long-term chronic infection tend to be resistant to more antibiotics than earlier or occasional isolates [16].

The variation in virulence factors and antibiotic resistance among *Achromobacter* spp. isolates highlights the necessity to better understand the involvement of these features in the pathogenic potential and mechanisms of colonization of these microorganisms. To this purpose, we evaluated virulence, cytotoxicity, biofilm formation and antibiotic susceptibility of clinical isolates causing occasional and chronic CF infections.

## 2. Results

Ninety-five *Achromobacter* spp. isolates were analyzed in this study. Seventy-nine isolates (range = 1–11 successively collected isolates, mean = 3.3 isolates/patient) were recovered from 24 chronically-infected CF patients with a mean time delay of 469 days (range = 21–1825 days). One isolate was recovered from each occasionally-infected CF patient (n = 14), except for two patients, P06 and P12, from whom we recovered 2 isolates with a time delay of 112 days and 155 days, respectively. We compared phenotypic features such as virulence, biofilm formation, cytotoxicity and antibiotic susceptibility between chronic and occasional and between early and late chronic isolates.

### 2.1. Virulence

**Figure 1:**
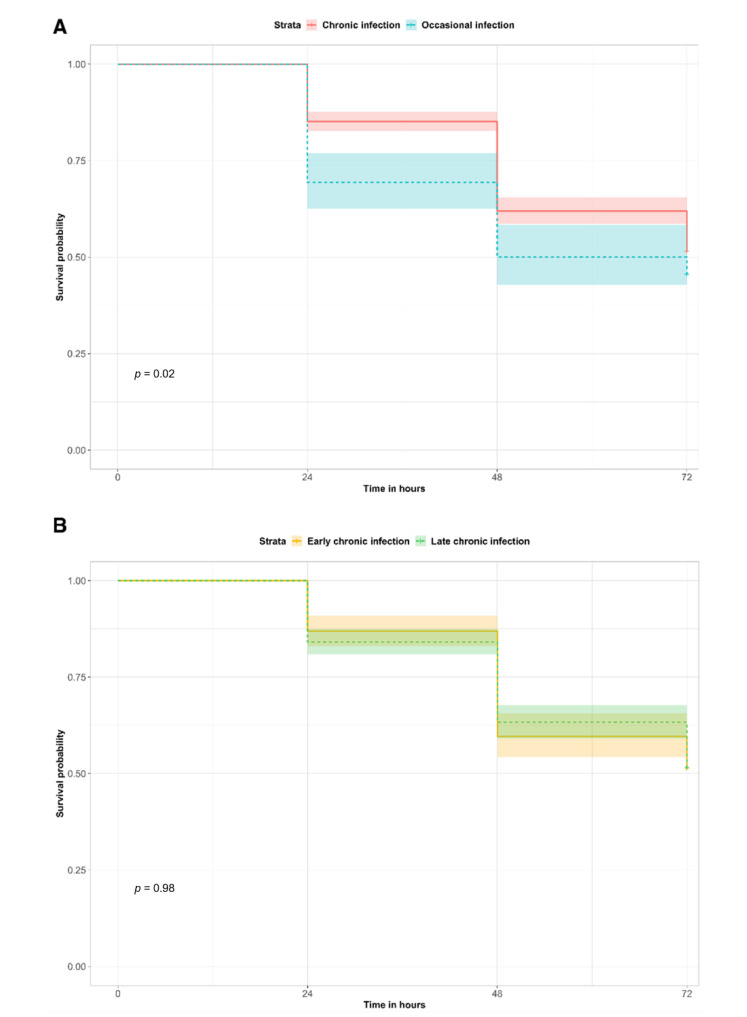
*G. mellonella* survival curve for virulence testing. The survival probabilities of *G. mellonella* larvae infected with chronic or occasional infection isolates (**A**) and with early or late chronic infection isolates (**B**) are reported at each time point. The *p*-values of the survival curve comparisons are indicated with *p*.

Virulence is an important feature for bacterial pathogenicity, invasion and interactions with the host. Virulence testing (Figure 1 and Figure S1) in a *G. mellonella* larvae model showed that isolates from occasionally-infected patients induced significantly higher mortality of larvae than chronic infection isolates (Kaplan–Meier survival estimate *p*-value = 0.02; Cox hazard ratio = 1.32; 95% CI = 1.04–1.66). When comparing early against late chronic isolates, no significant difference was observed.

**Figure 2:**
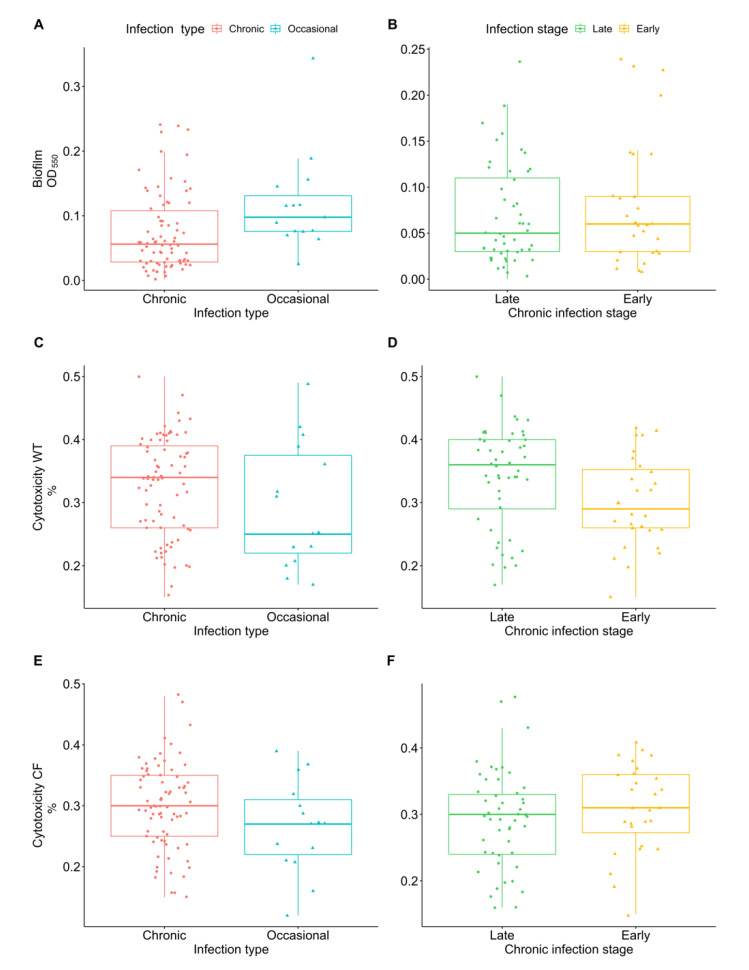
*Achromobacter* spp. biofilm formation and cytotoxicity on WT and CF bronchial epithelial cells. Biofilm formation in occasional and chronic isolates (**A**) and in early and late chronic isolates (**B**) measured by crystal violet staining (OD_550_). Cytotoxicity of occasional and chronic isolates and of early and late chronic isolates on WT cells (**C**,**D**) and CF cells (**E**,**F**) expressed as percentage of LDH release compared to the maximum value (positive control).

Biofilm formation plays an important role in the persistence of bacteria in CF chronic lung infections, protecting pathogens against environmental stress and increasing tolerance towards antibiotics and host defenses. No significant difference was observed in biofilm production among chronic and occasional isolates and between early and late chronic isolates (Figure 2A,B).

The ability to cause cytotoxicity could play an important role in tissue inflammation and degeneration. Cytotoxicity was assessed on both WT and CF bronchial epithelial cells. Although no statistically significant difference was found, we observed that chronic infection isolates showed a trend for greater cytotoxicity than occasional isolates in both cell types (Figure 2C,E). Moreover, when comparing early and late chronic isolates we observed an increase (Wilcoxon Mann–Whitney test *p*-value = 0.05 after 10,000 permutations) of cytotoxicity from early to late isolates in WT cells (Figure 2D).

Virulence, cytotoxicity and biofilm formation results per isolate are shown in Figure S2.

### 2.2. Antimicrobial Susceptibility

An important factor for the survival of infectious bacteria is their resistance to administered antibiotics, making infections hard to eradicate. We evaluated six antibiotics that have been reported to show variable susceptibility in chronic strains [16]: SXT, TGC, SSS, IPM, TZP and MEM.

**Figure 3:**
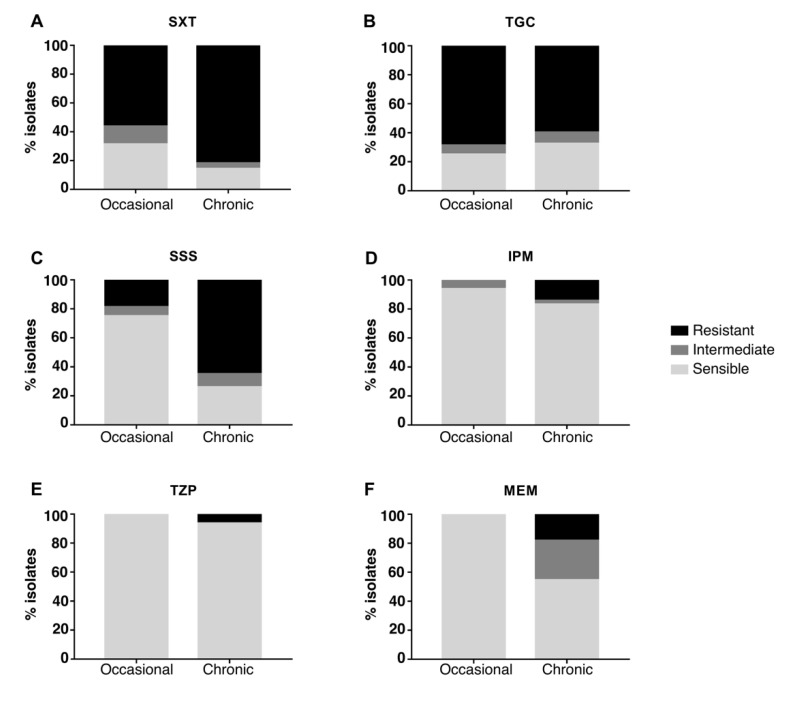
Antimicrobial susceptibility testing of chronic and occasional isolates. The percentage of resistant, intermediate and sensible isolates from chronic and occasional infection are represented for each antibiotic tested. SXT = trimethoprim–sulfamethoxazole (**A**), TGC = tigecycline (**B**), SSS = sulfonamides (**C**), IPM = imipenem (**D**), TZP = piperacillin-tazobactam (**E**), MEM = meropenem (**F**).

**Figure 4:**
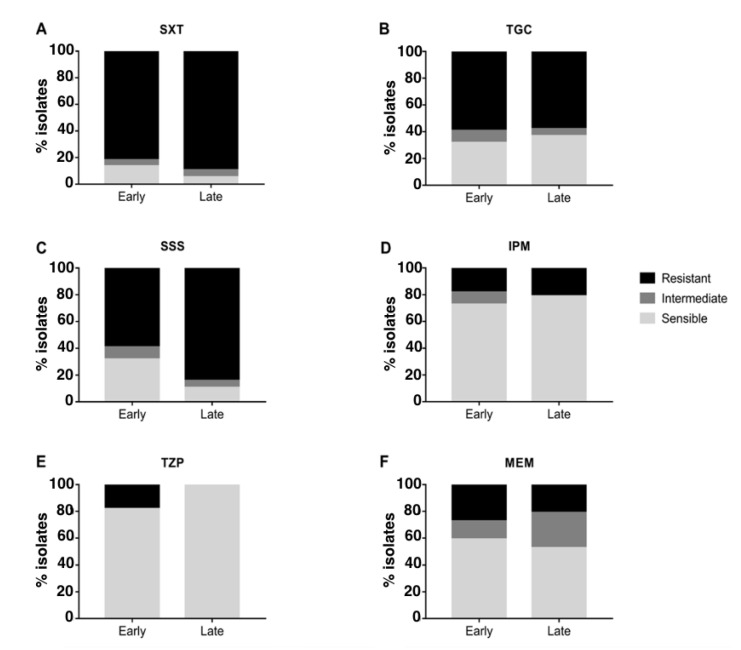
Antimicrobial susceptibility testing of early and late chronic isolates. The percentage of resistant, intermediate and sensible isolates from chronic and occasional infection are represented for each antibiotic tested. SXT = trimethoprim–sulfamethoxazole (**A**), TGC = tigecycline (**B**), SSS = sulfonamides (**C**), IPM = imipenem (**D**), TZP = piperacillin-tazobactam (**E**), MEM = meropenem (**F**).

The majority of strains were resistant to SXT and TGC and sensitive to IPM and TZP, while different susceptibility between occasional and chronic isolates was observed for SSS and MEM (Figure 3): when compared to occasional isolates, strains from chronically-infected patients were significantly more resistant to SSS (Fisher’s exact test *p*-value = 0.04 after 10,000 permutations; CI = 0.042–0.62; odds ratio = 0.17) and MEM (Fisher’s exact test *p*-value = 0.01 after 10,000 permutations; CI = 0–0.34; odds ratio = 0). No significant difference in resistance was observed when comparing early and late chronic isolates (Figure 4). Antimicrobial susceptibility results per isolate are shown in Figure S3.

### 2.3. Biomarkers of Antimicrobial Resistance

Genome analysis of the 54 *Achromobacter* spp. isolates from the Verona collection was performed to identify genetic components significantly associated with virulence, biofilm, cytotoxicity and antimicrobial resistance. As regards the former three features, no genetic loci were significantly associated to the phenotypic traits. Within the tested antibiotics, we found various candidate biomarkers of resistance or sensitivity linked to transmembrane transporters and efflux pumps (e.g., secretory components, ABC transporters), transcriptional regulators (e.g., AraC) and metabolic enzymes (Table 1 and Table S1 for details). In addition, some hypothetical proteins were associated with sensitivity to SSS and resistance to MEM. Some node sequences showed statistically significant correlation (*q*-value ≤ 0.05) with both IPM and MEM, and with both SSS and SXT (Table S1).

## 3. Discussion

To identify phenotypic features related to the ability of *Achromobacter* spp. to establish chronic or occasional colonization in CF airways, we evaluated virulence, biofilm formation, cytotoxicity, and antimicrobial susceptibility of 95 clinical isolates and compared results between chronic and occasional isolates and between early and late chronic ones.

Isolates were collected at the CF Center of Verona (Italy) and Bambino Gesù Hospital in Rome (Italy). While the Verona collection included a larger number of occasional isolates, the Rome one comprised many longitudinally-isolated chronic strains encompassing a long period of time. The two collections showed similar phenotypic characteristics (Figures S2 and S3); so, their combination allowed the analysis of a more homogeneous group of strains than using them separately; however, the final collection still included a lower number of occasional (*n* = 16) than chronic isolates (*n* = 79, 30 early and 49 late chronic isolates).

To evaluate the virulence of *Achromobacter* spp. isolates, we used the well-characterized *G. mellonella* larvae model [17,18]. Occasional infection isolates caused higher mortality than strains from chronic infection, indicating that *Achromobacter* spp. exhibit higher virulence during occasional infection. This observation suggests that virulence attenuation could be a key factor during the establishment of chronic infection. Differences in virulence between early and late chronic strains of another CF pathogen, *Pseudomonas aeruginosa,* were also previously highlighted [18], leading to the hypothesis that multiple mutations could be responsible for virulence attenuation during late infection. Although we did not observe a significant difference in virulence between early and late chronic isolates of *Achromobacter* spp., a similar mechanism of adaptation could be proposed for occasional and chronic isolates, where selection of strains with an increasing ability to persist may occur.

As for biofilm formation, the majority of our isolates showed a low-moderate production of biofilm, confirming the poor adhesion ability of *Achromobacter* spp. on surfaces [7,9]. Although no significant difference in biofilm production was observed between chronic and occasional isolates nor between early and late chronic isolates, the great majority of strains unable to form biofilm were isolated from chronic infection. This could suggest a mechanism of within-host adaptation in the CF lung; e.g., acquisition of mutations in genes with a role in surface adhesion could lead to decreased biofilm production or to formation of unattached aggregates [10,13,19].

To investigate whether a reduced virulence in the chronic isolates coincided with lower cytotoxicity, we compared the cytotoxic potential of chronic and occasional strains in WT and F508del human bronchial epithelial cultured cells, but no significant difference was found. Although no statistically significant difference was found, we observed that chronic infection isolates induced slightly greater cytotoxicity than occasional isolates—an opposite trend compared to the results of biofilm and virulence testing. We observed an increased cytotoxicity from early to late isolates in cells expressing WT CFTR. No significant difference was observed in CF cells; this could indicate an underlying adaptation of late chronic strains to the CF lung environment leading to a more indolent colonization.

Biomarkers analysis of virulence traits (biofilm, cytotoxicity and virulence in *G. mellonella* larvae) showed no associated genetic loci. This is probably due to the fact these aspects are known to be mainly regulated through RNA modulation [20,21] rather than through the accumulation of genomic mutations.

*Achromobacter* spp. are reported to increasingly develop resistance to various antibiotics. We tested susceptibility of all isolates to 6 antibiotics that have been reported to show variable susceptibility in chronic strains [16]. *Achromobacter* spp. strains generally displayed resistance to TGC and susceptibility to TZP and IPM, confirming their previously-reported innate resistance to tetracyclines [2] and susceptibility to beta-lactams and carbapenems [16]. Interestingly, an increased resistance to MEM was observed in chronic isolates. Moreover, even though IPM and MEM belong to the same class of antimicrobials, we observed both imipenem resistant but meropenem susceptible (IRMS) and meropenem resistant but imipenem susceptible (MRIS) phenotypes. These phenotypes were previously reported for *P. aeruginosa* and various members of the *Enterobacteriaceae* family [22] as well as for *Achromobacter* spp. clinical isolates [22,23]. In addition, we observed significantly higher resistance to SSS in chronic infection isolates than in occasional ones, in concordance with previous studies [23,24]. Resistance of chronic infection isolates to SSS could be associated with the use of SXT to eradicate CF pathogens. The majority of both occasional and chronic isolates was resistant also to SXT, in contrast with previous investigations; e.g., a recent one found that the majority of *Achromobacter* spp. strains from Danish CF patients were sensitive to SXT [16], considered as one of the most active agents against *Achromobacter* spp. infections [25]. This suggests that the acquisition of resistance to SXT might have occurred among isolates in our collections, e.g., through the spread of mobile genetic elements carrying resistance genes [26] or due to the different antibiotic treatment regimens used.

Candidate biomarkers were identified for SXT, IPM, MEM and SSS by analyzing sequenced isolates. Some of them are known to be involved in antibiotic resistance, such as ABC transporters [27], while the role of other candidates—associated with antibiotic resistance or with sensitivity—should be further investigated. Of particular interest are hypothetical proteins, which could provide additional information on *Achromobacter* spp. resistance mechanisms upon further characterization. These candidate biomarkers could help in the identification of strains that are becoming persistent and support their eradication before chronic infection is fully developed. Finally, in order to assess whether biomarkers presence could be linked to an adaptation mechanism such as clonal expansion or horizontal gene transfer, we further performed genome analysis but neither of these hypotheses was confirmed. In conclusion, our results show that *Achromobacter* spp. isolates from chronic and occasional lung infection exhibit different virulence and antibiotic resistance characteristics, some of which might be linked to persistence in CF lungs. We identified potential predictive markers of persistence such as decreased virulence, higher cytotoxicity, resistance to antibiotics, as well as genetic biomarkers [3], that could be translated into the clinical setting either to help preventing the development of chronic infections or to support therapeutic treatments aimed at eradicating *Achromobacter* spp.

## 4. Materials and Methods

### 4.1. Samples Collection

Ninety-five *Achromobacter* spp. isolates were collected from the sputum samples of 38 patients followed at the CF Center of Verona and Bambino Gesù Hospital in Rome (Italy): 54 isolates were recovered from 26 patients in Verona [3,26], while 41 isolates were recovered from 12 patients in Rome. Patients were classified as occasionally- and chronically-infected with *Achromobacter* spp. according to the European Consensus Criteria or Leeds criteria. In the Verona collection, 43 longitudinal isolates were collected from 17 patients with chronic infections while 11 strains were collected from 9 patients with occasional infection. The Rome collection comprised 36 strains collected over time from 7 patients with chronic infection and 5 strains collected from 5 patients with occasional infections. Isolates from chronically-infected patients were further classified as early (<1 year from 1st colonization event) and late isolates (>1 year from 1st colonization). Relatedness of strains was verified to confirm that subsequent isolates from one patient actually represented a single strain. An overview of the isolates included in each collection is reported in Table 2. Informed consent was obtained according to projects CRCFC-CEPPO026 and CRCFC-CEPPO031, approved by the Ethical Committee. All the isolates included in this study were identified as *Achromobacter* spp. by MALDI-TOF-MS (bioMerieux Marcy-l’Étoile, France). Strains were stored in Microbank (Pro-Lab Diagnostics, Neston, UK) at −80 °C. Detailed information regarding the collections is reported in Table S2.

### 4.2. Virulence Testing

Virulence was assessed in *Galleria mellonella* larvae. Ten larvae were inoculated with a 1 × 10^6^ CFU bacterial suspension of each clinical isolate through the last proleg into the haemocoel using a 0.3 mL syringe and incubated in Petri dishes, on filter paper, at 37 °C, in the dark. In the control group, larvae were injected with sterile saline solution. Larvae were monitored daily up to 72 h and death was assessed by lack of movement after stimulation and blackening.

### 4.3. Biofilm Formation Assay

Bacterial strains were plated onto LB agar and grown at 37 °C for 24–48 h. A single colony was inoculated in BHI medium and grown for 16 h at 37 °C with shaking. OD_600_ was measured, cultures were diluted to 0.1 OD/mL and 200 µL/well were incubated in a 96-well plate for 24 h at 37 °C. Wells were washed with saline solution and stained with 0.1% crystal violet solution for 15 min, then rinsed, washed with water and air dried. After 30 min of incubation with 30% acetic acid at 37 °C, absorbance at 550 nm was measured.

### 4.4. Cytotoxicity Testing

Human CF bronchial epithelial cell lines CFBE14o- 4.7 WT-CFTR (WT cells) and DeltaF508-CFTR (CF cells) (Merck, Darmstadt, Germany), overexpressing WT and F508del CFTR cDNA, respectively, were cultured in 200 µL EMEM supplemented with 1% Fetal Bovine Serum, 0.5–2 µg/mL Puromycin and 2 mM L-Glutamine into Fibronectin/Collagen/BSA-coated 96-well plates incubated at 37 °C and 5% CO_2_. At 80% confluency, 50 µL/well of 2 OD_600_/mL bacterial suspension were added to cell cultures and incubated at 37 °C for 4 h. CytoTox 96 Non-Radioactive Cytotoxicity Assay (Promega) measuring LDH release was used according to manufacturer’s instructions. Briefly, 50 µL of cells suspension were added with 50 µL of CytoTox 96 Reagent and incubated for 30 min in the dark. After adding 50 µL of Stop Solution, absorbance at 450 nm was recorded. Cytotoxicity was calculated by dividing for the absorbance of the positive control (treated with Lysis Solution).

### 4.5. Antimicrobial Susceptibility Testing

Antimicrobial susceptibility was determined by disk diffusion assay. Bacterial suspension at 0.5 McFarland was streaked onto Mueller–Hinton agar plates, antibiotic-containing disks (Oxoid) were placed onto the agar surface, plates were incubated at 37 °C for 48 h and the diameter of the zone-of-inhibition was measured. Disks contained 1.25/23.75 µg trimethoprim–sulfamethoxazole (SXT), 15 µg tigecycline (TGC), 300 µg sulfonamides (sulphadiazine, sulphathiazole) (SSS), 10 µg imipenem (IPM), 100/10 µg piperacillin-tazobactam (TZP) or 10 µg meropenem (MEM). Since no EUCAST or CLSI breakpoint standard is available for *Achromobacter* spp., susceptibility profiles were interpreted as resistant (R), intermediately resistant (I) or sensible (S) based on breakpoints proposed in previous literature [15,16,28].

### 4.6. Statistical Analysis

Statistical analysis was carried out to compare chronic and occasional isolates and early and late chronic isolates. Virulence results were tested by the Kaplan–Meier method using the log-rank test to compare the overall survival of larvae over an observation period of 72 h. Hazard ratios were computed with a Cox regression model. Cytotoxicity and biofilm formation results were tested using a Wilcoxon Mann–Whitney test. Outlier values were observed in biofilm formation results (*n* = 2, isolates 7-3 and 12-2) and were excluded from statistical analysis. Fisher’s exact test was used to ascertain the significance of antibiotic susceptibility results. Since observations per isolate were not independent due to a longitudinal collection strategy, for each test, *p*-values were adjusted performing 10,000 permutations of the infection type (chronic, occasional) or infection stage (early chronic, late chronic) stratified by collection (Verona, Rome). R version 4.0.4 (R Foundation for Statistical Computing, Vienna, Austria) [29] was used for statistical analysis and for results visualization. Boxplots were generated using ggpubr v0.4.0, survival curves using survminer v0.4.9, and heatmaps using pheatmap v1.0.8 R libraries.

### 4.7. Identification of Genetic Loci Associated with Virulence, Biofilm, Cytotoxicity and Antimicrobial Resistance

The DBGWAS 0.5.4 software (bioMerieux, Lyon, France) [30] was used to identify genetic components significantly associated with virulence, biofilm, cytotoxicity and antimicrobial resistance. In previous work [3,26] we sequenced the whole genome of the 54 *Achromobacter* spp. isolates from the Verona collection. A phylogenetic analysis and a comparison of virulence and resistance genes, genetic variants and mutations, and hypermutability mechanisms between chronic and occasional isolates was also performed. The de novo assembled contigs of these genomes were used as input for the association analysis. The following phenotypic cut-offs were used as parameters when running the analysis: biofilm production of 0.115 (absorbance at 550 nm), cytotoxicity of 0.36 (% cytotoxicity vs. positive control), virulence causing 5 dead larvae in 48 h; while the cut-offs for antimicrobial resistance were defined as described in Section 4.5. In particular, DBGWAS accepts continuous phenotypes, so we translated the categorical variables “resistant (R)”, “intermediately resistant (I)” and “sensible (S)” to a dummy variable such that S = 0, I = 1 and R = 2. All available annotations of *Achromobacter* genes from the UniProt database (www.uniprot.org, ref. 320,589 genes; accessed on 1 March 2022) were used in the annotation step of the virulence, biofilm and cytotoxicity association analysis, whereas all known bacterial resistance genes from the UniProt database (www.uniprot.org, ref. 36,658 genes; accessed on 1 March 2022) were used for annotation in the antimicrobial resistance association analysis.

Sensitivity and specificity of candidate components having the same order of magnitude of the lowest *q*-value—i.e., *p*-value adjusted for the False Discovery Rate—of each analysis were calculated using the following formulas: for nodes positively associated to the phenotype sensitivity = Pheno1Count/Pheno1TotalCount and specificity = (Pheno0TotCount-Pheno0Count)/Pheno0TotCount; for nodes negatively associated to the phenotype sensitivity = Pheno0Count/Pheno0TotCount and specificity = (Pheno1TotCount-Pheno1Count)/Pheno1TotCount. In particular, Pheno1Count = isolates displaying the phenotype and carrying the allele, Pheno1TotalCount = isolates displaying the phenotype, Pheno0Count = isolates not displaying the phenotype and carrying the allele, Pheno0TotCount = isolates not displaying the phenotype. All candidate components having the highest sensitivity and specificity, down to a threshold of 80%, and lowest *q*-value were further analyzed (tests were considered to be statistically significant if *q*-value < 0.05). Every k-mer present in only one isolate was discarded.

## Figures and Tables

**Table 1:** Genetic loci significantly associated with antimicrobial resistance. The most significant results of association analysis are reported. IPM = imipenem, SXT = trimethoprim–sulfamethoxazole, SSS = sulfonamides (sulphadiazine, sulphathiazole), MEM = meropenem, *q*-value = *p*-value adjusted for the False Discovery Rate.

Antimicrobial	Associated with	Node ID	Sensitivity (%)	Specificity (%)	*q*-Value	Annotation
IPM	Resistance	n1316743	100.0	100.0	8.4 × 10^−23^	Arginine-tRNA ligase
IPM	Resistance	n284357	100.0	100.0	8.4 × 10^−23^	Diguanylate cyclase (GGDEF domain) with GAF sensor
IPM	Resistance	n1001723	100.0	100.0	8.4 × 10^−23^	ABC transporter
IPM	Resistance	n1228398	100.0	100.0	8.4 × 10^−23^	Type II secretory pathway component GspD
IPM	Resistance	n1320154	100.0	100.0	8.4 × 10^−23^	AraC family transcriptional regulator
IPM	Resistance	n119911	100.0	100.0	8.4 × 10^−23^	NA
IPM	Resistance	n1360642	100.0	100.0	8.4 × 10^−23^	General secretion pathway protein GspN
IPM	Resistance	n1607259	100.0	100.0	8.4 × 10^−23^	Fe^2+^-dicitrate sensor, membrane component, FecR
IPM	Resistance	n1496359	100.0	100.0	8.4 × 10^−23^	Glutathione S-transferase family protein
MEM	Resistance	n382985	88.9	97.1	1.3 × 10^−2^	ABC transporter ATP-binding protein
MEM	Resistance	n776344	88.9	97.1	1.3 × 10^−2^	Hypothetical protein
MEM	Resistance	n1454097	88.9	97.1	1.3 × 10^−2^	ABC transporter ATP-binding protein
SSS	Susceptibility	n477893	81.3	86.1	2.8 × 10^−3^	Hypothetical protein
SSS	Susceptibility	n1248808	81.3	86.1	2.8 × 10^−3^	ABC transporter ATP-binding protein
SSS	Susceptibility	n888310	81.3	86.1	2.8 × 10^−3^	Aminomethyl-transferring glycine dehydrogenase
SXT	Susceptibility	n1221225	85.7	100.0	7.6 × 10^−9^	NA
SXT	Susceptibility	n1593088	85.7	100.0	7.6 × 10^−9^	16S rRNA (uracil(1498)-N(3))-methyltransferase
SXT	Susceptibility	n222900	85.7	100.0	7.6 × 10^−9^	NA
SXT	Susceptibility	n979447	85.7	100.0	7.6 × 10^−9^	DNA mismatch repair endonuclease MutL
SXT	Susceptibility	n120539	85.7	100.0	7.6 × 10^−9^	Efflux RND transporter periplasmic adaptor subunit
SXT	Susceptibility	n335885	85.7	100.0	7.6 × 10^−9^	Efflux RND transporter periplasmic adaptor subunit
SXT	Susceptibility	n71145	85.7	100.0	7.6 × 10^−9^	M61 family metallopeptidase
SXT	Susceptibility	n346933	85.7	100.0	7.6 × 10^−9^	Acyl-CoA synthetase
SXT	Susceptibility	n1069885	85.7	100.0	7.6 × 10^−9^	Helix-turn-helix domain-containing protein
SXT	Susceptibility	n1157928	85.7	100.0	7.6 × 10^−9^	Helix-turn-helix domain-containing protein
SXT	Susceptibility	n527920	85.7	100.0	7.6 × 10^−9^	Patatin-like phospholipase family protein

**Table 2:** *Achromobacter* spp. collections summary. The number of isolates included in each collection is reported in the table; the number of patients from which the strains were collected is indicated in parenthesis. E = early chronic infection isolates; L = late chronic infection isolates.

	Rome-*n* (Patients)		Verona-*n* (Patients)	
Chronic infection isolates	36 (7)	E: 6 (4)	43 (17)	E: 24 (10)
		L: 30 (7)		L: 19 (10)
Occasional infection isolates	5 (5)		11 (9)	

## Data Availability

Sequences of the 54 *Achromobacter* spp. isolates from the Verona collection have been deposited at the NCBI SRA database under project n. PRJEB40979.

## Supplementary Materials

The following supporting information can be downloaded at: https://www.mdpi.com/article/10.3390/ijms23169265/s1.
