# Supplementary material for: Achromobacter spp. Adaptation in Cystic Fibrosis Infection and Candidate Biomarkers of Antimicrobial Resistance
Source: Int J Mol Sci. 2022 Aug 17;23(16):9265. doi: 10.3390/ijms23169265 (PMC9409262; doi:10.3390/ijms23169265)
Supplement: Supplementary file 1 [file ijms-23-09265-s001.zip › 06-Supplementary_S1_S2_S3.pdf]

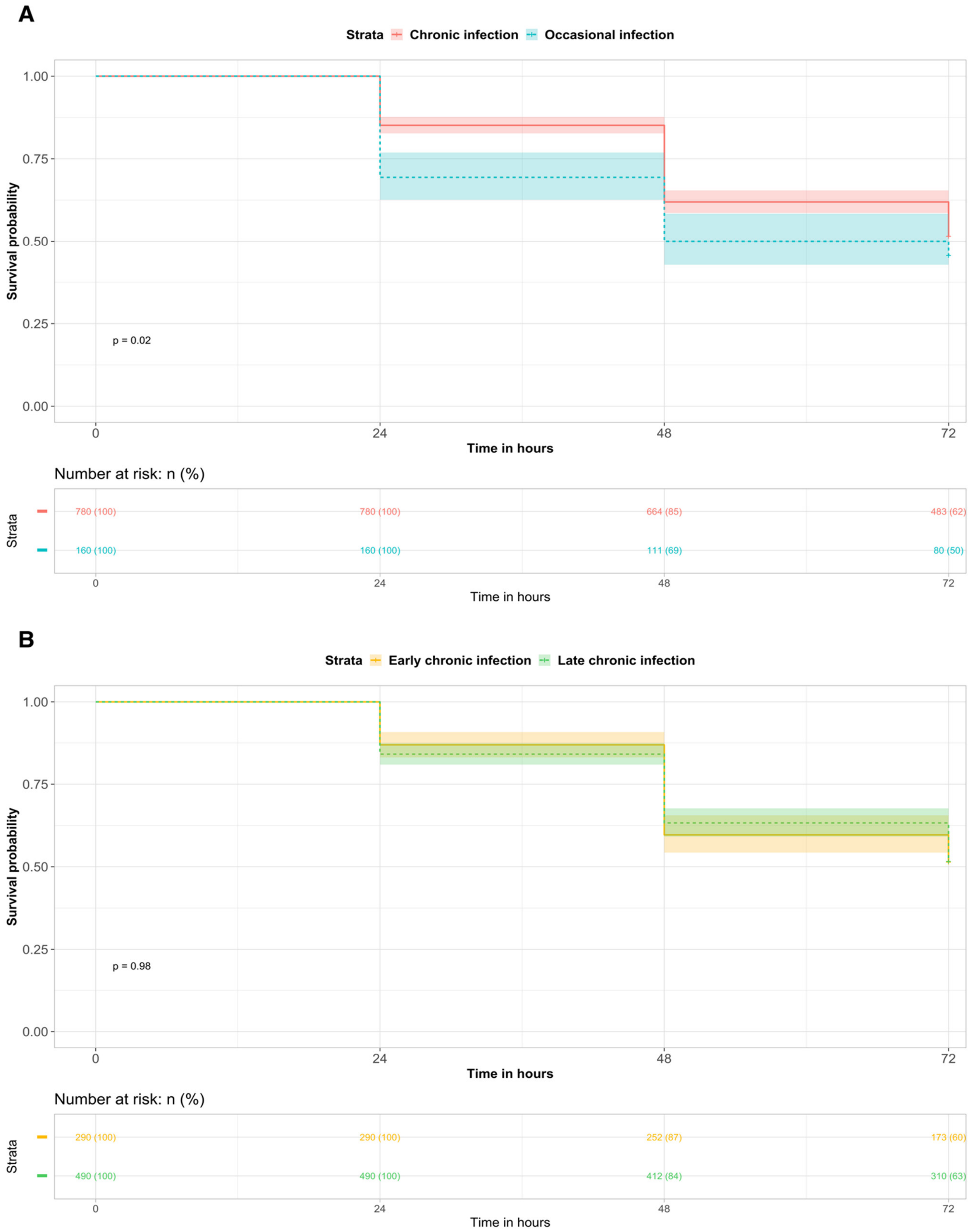

Figure S1. *Galleria mellonella* survival curve for virulence testing. The survival probabilities of *G. mellonella* larvae infected with chronic or occasional infection isolates (**A**) and with early or late chronic infection isolates (**B**) are reported at each time point. The p-values of the survival curves comparisons are indicated with p. Risk tables indicating the absolute number and the percentage (between parenthesis) of *G. mellonella* larvae at risk by time for each group (strata) are also reported.

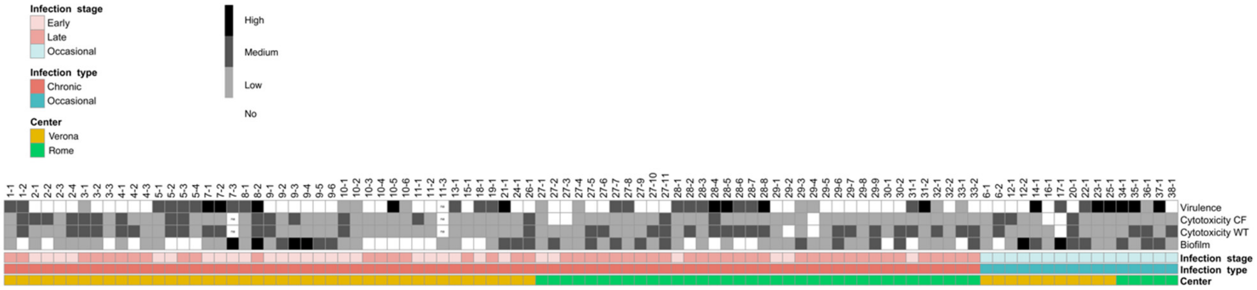

Figure S2. Virulence, cytotoxicity in WT and CF cells, and biofilm formation results per isolate. Each feature was defined as high, medium, low or none as follows. Biofilm was classified as proposed by Stepanovic and colleagues (2007, doi:10.1111/j.1600-0463.2007.apm_630.x). Cytotoxicity's lower cut-off was calculated as SD above the mean OD of the blank; 2- and 4-folds values were used to discriminate between the other levels. Virulence was defined from high to low with at least 50% larvae mortality within 24, 48 and 72 hours respectively and none with less than 50% mortality at 72 hours. na=not available.

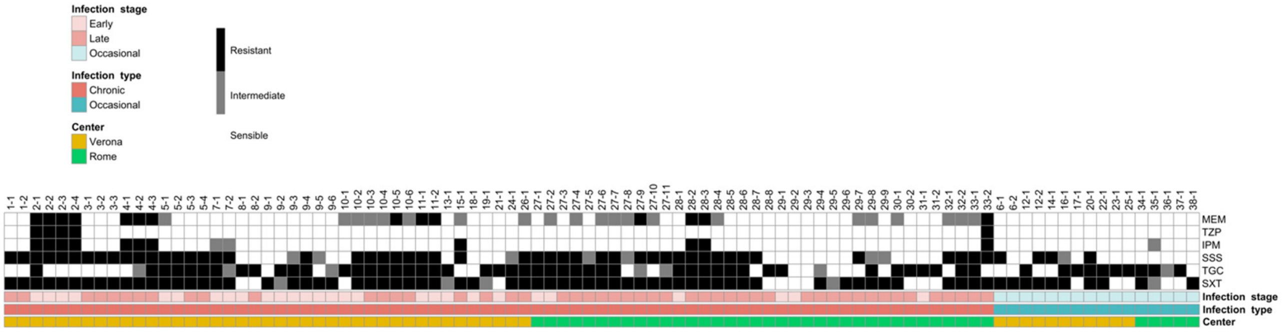

Figure S3. Antimicrobial susceptibility results per isolate. Susceptibility to antibiotics was tested by Kirby-Bauer disk diffusion test (SXT=trimethoprim-sulfamethoxazole, TGC=tigecycline, SSS=sulfonamide, IPM=imipenem; TZP=piperacillin-tazobactam, MEM=meropenem).
